# Iodinated contrast agents in patients with myasthenia gravis: a retrospective cohort study

**DOI:** 10.1007/s00415-017-8518-8

**Published:** 2017-05-26

**Authors:** Jakob Rath, Matthias Mauritz, Gudrun Zulehner, Eva Hilger, Hakan Cetin, Gregor Kasprian, Eduard Auff, Fritz Zimprich

**Affiliations:** 1 Department of Neurology, Medical University of Vienna, Währinger Gürtel 18-20, 1090 Vienna, Austria; 2 Department of Biomedical Imaging and Image-guided Therapy, Medical University of Vienna, Währinger Gürtel 18-20, 1090 Vienna, Austria

**Keywords:** Iodinated contrast agent, Myasthenia gravis, Computed tomography, Adverse events, Myasthenic crisis, Anaphylaxis

## Abstract

Currently, it has not been satisfactorily established, whether modern low-osmolality iodinated contrast agents (ICAs) used in computed tomography (CT) studies are a risk factor for exacerbation of myasthenic symptoms. The rate of acute adverse events as well as delayed clinical worsening up to 30 days were analyzed in 73 patients with confirmed myasthenia gravis (MG) who underwent contrast-enhanced CT studies and compared to 52 patients who underwent unenhanced CT studies. One acute adverse event was documented. 12.3% of MG patients experienced a delayed exacerbation of symptoms after ICA administration. The rate of delayed severe exacerbation was higher in the contrast-enhanced group. Alternative causes for the exacerbation of MG-related symptoms were more likely than ICA administration in all cases. ICA administration for CT studies in MG patients should not be withheld if indicated, but patients particularly those with concomitant acute diseases should be carefully monitored for exacerbation of symptoms.

## Introduction

Myasthenia gravis (MG) is an autoantibody-mediated autoimmune disease of the neuromuscular junction characterized by muscle weakness and abnormal fatigability. Prevalence rates of around 16 per 100,000 [1] mean all branches of medicine care for patients with myasthenia gravis. One feared complication of myasthenia gravis is a clinical deterioration upon a wide range of reported drugs. While the risk posed by some medications (e.g., quinolones) is well documented [2], conflicting results have been published regarding the exacerbation of myasthenic symptoms following the administration of iodinated contrast agents (ICAs) used for computed tomography (CT) [3, 4].

ICAs can be divided by their osmolality. Older substances were of very high osmolality (above 1500 mOsm/L) and are no longer used routinely because of their comparatively high toxicity. Modern ICAs, in contrast, are low-osmolality agents (290–860 mOsm/L) and generally better tolerated [5]. MG-unrelated acute adverse events (e.g., anaphylactoid reactions, contrast-induced nephropathy, or extravasation of contrast agent) were estimated to occur in up to 3% of all patients receiving low-osmolality contrast agents, though only a small fraction of these are classified as severe [6]. Delayed adverse events (predominately cutaneous reactions appearing up to 1 week after administration) are estimated by some to be as high as 14% [7].

Myasthenic crisis following the administration of old, high-osmolality ICAs was initially reported in the 1980s in a number of case reports [8–13]. One systematic retrospective study on acute adverse events (within 24 h) associated with high-osmolality agents presented five patients with a subsequent exacerbation of their myasthenic weakness, although in each case, alternative explanations for the deterioration other than ICA administration were possible [14].

Immediate adverse reactions including myasthenic exacerbations were reported in less than 1% of patients in one recent study on low-osmolality ICAs [4]. Another study on low-osmolality agents observed an exacerbation rate of 12.5% in MG patients within 45 days of ICA administration [3].

In summary, there are still too few data available to confidently judge the risk that modern ICAs pose for patients with myasthenia gravis, especially with regard to a delayed exacerbation of myasthenic symptoms. Since ICAs are frequently required in many different indications in modern day medicine, there is an urgent need to better evaluate this risk. This point was recently stressed by a report on this issue by the Myasthenia Gravis Foundation of America (MGFA), which called for more data to be generated on this topic [2].

The aim of this study was to add more information on this question by retrospectively examining adverse event rates and delayed exacerbations of myasthenic symptoms after administration of modern low-osmolality ICAs in patients with confirmed myasthenia gravis.

## Materials and methods

### Patients

426 consecutive patients (186 male, 240 female) with confirmed myasthenia gravis who were treated at the Department of Neurology of the Medical University of Vienna were retrospectively identified and considered for inclusion into this study. All patients were required to meet the standard diagnostic criteria consisting of the typical clinical symptoms in combination with either a positive test for myasthenia gravis-specific autoantibodies [acetylcholine receptor or muscle-specific kinase (MuSK)], a typical decrement (>10%) shown by repetitive nerve stimulation or a positive edrophonium chloride test.

**Fig. 1:**
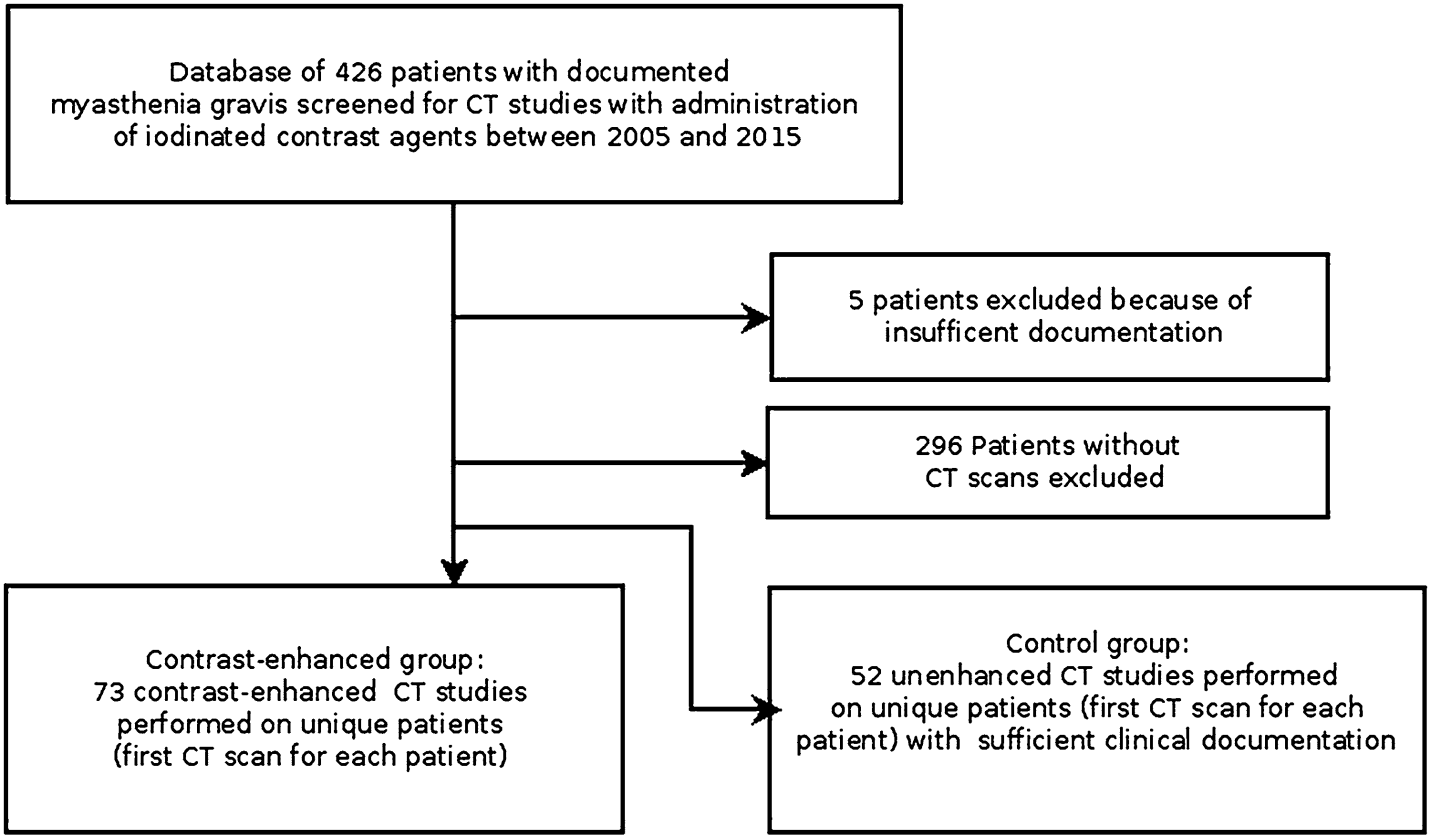
Flowchart of study population. *CT* computed tomography

By reviewing the medical charts of these patients, we next identified 73 (31 male, 42 female, and median age 62) who underwent CT scans with the administration of low-osmolality ICAs at the Department of Biomedical Imaging and Image-guided Therapy within our hospital between 2005 and 2015. A non-overlapping control group of 52 patients (25 male, 27 female, and median age 64) consisted of patients with myasthenia gravis who underwent only unenhanced CT studies during the same period (Fig. 1). Only one (i.e., the first) CT scan was considered for each patient to avoid double inclusions into any of the study groups. CT studies were, furthermore, only included if a sufficient clinical documentation was available to assess the clinical state of patients before and for 30 days after the CT study. Exclusion criteria were congenital myasthenia gravis, concomitant serious renal disease, and an age of less than 18 years. Ethical approval was obtained from the institutional ethics committee and the requirement to obtain patient consent was waived for this retrospective study.

### Data analysis

Baseline demographic and all clinical characteristics were retrospectively extracted from medical records (Table 1). Baseline MGFA state was defined as MGFA class at the time of CT study. The primary endpoint was defined as a clinically relevant deterioration of myasthenic symptoms within 30 days of the CT study, defined as clinical worsening by at least one MGFA class. Subgroup analysis was performed for patients with an increase of the MGFA state by at least one class but not fulfilling the criteria of myasthenic crisis (i.e., MGFA class V) and for patients with a severe worsening defined by the occurrence of a myasthenic crisis with respiratory insufficiency and intubation or death within the post-scan observation period. Secondary endpoints were (a) the occurrence of an immediate, acute adverse reaction as documented in the radiological report (b) in the case of reaching the primary endpoint the time (in days) to clinical deterioration after ICA administration.

**Table 1:** Baseline characteristics

CT study	All patients			Patients reaching primary endpoint	
	Contrast-enhanced CT scans, *N* = 73	Controls (unenhanced CT scans), *N* = 52	*P* value*	Contrast-enhanced CT scans, *N* = 9	Controls (unenhanced CT scans), *N* = 2
Sex			0.534		
Male	31 (42.5%)	25 (48.1%)		2 (22.2%)	1 (50%)
Female	42 (57.5%)	27 (51.9%)		7 (78.8%)	1 (50%)
Age (median)	62 (range 79)	64 (range 77)	0.389	69 (range 21)	64 (range 0)
Antibodies			0.679		
AChR	60 (82.2%)	40 (76.9%)		9 (100%)	2 (100%)
MuSK	2 (2.7%)	2 (3.8%)		0	0
Negative	10 (13.7%)	10 (19.2%)		0	0
No data	1 (1.4%)	0		0	0
Disease duration (median)	18 months (range 486)	24 months (range 528)	0.755	5 months (range 486)	1 months (range 2)
MGFA class			0.404		
CSR/PR/MM	11 (15.1%)	13 (25%)		1 (11.1%)	0
1	14 (19.2%)	12 (23.1%)		0	0
2	23 (31.5%)	17 (32.7%)		1 (11.1%)	1 (50%)
3	20 (27.4%)	7 (13.5%)		5 (55.6%)	1 (50%)
4	3 (4.1%)	1 (1.9%)		1 (11.1%)	0
5	2 (2.7%)	2 (3.8%		1 (11.1%	0
Thymectomy	37 (50.7%)	19 (36.5%)	0.117	3 (33.3%)	0
Thymic pathology			0.442		
Normal	10 (13.7%	3 (5.8%)		2	0
Hyperplasia	9 (12.3%)	6 (11.5%)		0	0
Thymoma	12 (16.4%	8 (15.4%)		1	0
No histology	6 (8.2%)	2 (3.8%)		0	0
Therapy					
No Therapy	12 (16.4%)	10 (19.2%)	0.686	3 (33.3%)	0
Cholinesterase inhibitor	58 (79.5%)	38 (73.1%)	0.405	6 (66.7%)	2 (100%)
Cortisone	29 (39.7%)	21 (40.44%)	0.941	0	0
Immunosuppressant	20 (27.4%)	8 (15.4%)	0.112	1 (11.1%)	0
IVIG	1 (1.4%)	4 (7.7%)	0.159	1 (11.1%)	1 (50%)
Plasma exchange	8 (11%)	4 (7.7%)	0.76	1 (11.1%)	1 (50%)
Repetitive nerve stimulation			0.814		
Positive	33 (45.2%)	22 (42.3%)		5 (55.6%)	1 (50%)
Negative	20 (27.4%)	13 (25%)		2 (20.2%)	1 (50%)
No data	20 (27.4%)	17 (32.7%)		2 (20.2%)	0
Edrophonium test			0.915		
Positive	37 (50.7%)	28 (53.8%)		5 (55.6%)	1 (50%)
Negative	10 (13.7%)	6 (11.5%)		1 (11.1%)	1 (50%)
No data	26 (35.6%)	18 (34.6%)		3 (33.3%)	0
Concomitant acute diseases at CT			0.038^‡^		
None	46 (63%)	44 (84.6%)		3 (33.3%)	2 (100%)
Cardiac	2 (2.7%)	2 (3.8%)		1 (11.1%)	
Pulmonary (non-neuromuscular)	6 (8.2%)	0		2 (22.2%)	
Neurological (not MG related)	1 (1.4%)	1 (1.9%)			
Other	18 (24.7%)	5 (9.6%)		3 (33.3%)	
Indication			<0.000^‡^		
Thymus evaluation	31 (42.5%)	19 (36.5%)		2 (22.2%)	2 (100%)
Focal neurological symptoms	7 (9.6%)	12 (23.1%)		1 (11.1%)	0
Dyspnea	12 (16.4%)	0		1 (11.1%)	0
Acute non-neurological symptoms	21 (28.8%)	4 (7.7%)		5 (55.6%)	0
Chronic disease/symptoms	2 (2.7%)	8 (15.4%)		0	
Trauma	0	9 (17.3%)		0	0
Region			<0.000^‡^		
Chest	41 (56.2%)	22 (42.3%)		6 (66.7%)	2 (100%)
Abdomen	11 (15.1%)	3 (5.8%)		1 (11.1%)	0
Chest/abdomen	11 (15.1%)	0		2 (22.2%)	0
Head/CT angiography	5 (6.8%)	17 (32.7%)		0	0
Other	5 (6.8%)	10 (19.2%)		0	0

Baseline characteristics of all patients and of those who reached the primary endpoint

*AChR* acetylcholine receptor, *MuSK* muscle-specific tyrosine kinase, *IVIG* intravenous immunoglobulin, *SD* standard deviation, *ICA* iodinated contrast agent, *CSR* complete stable remission, *PR* pharmacologic remission, *MM* minimal manifestation, *NA* not applicable

* *P* values were obtained with the Mann–Whitney *U* or Student’s *t* test (for continuous variables) and with the Fisher’s exact test or Chi-square test (for categorical variables) as appropriate

^‡^Statistically significant

Statistical analysis was performed with the SPSS 22 software package (IBM Corp. Released 2013. IBM SPSS Statistics for Macintosh, Version 22.0. Armonk, NY: IBM Corp). Baseline variables between the two groups of patients were compared using the Mann–Whitney *U* or Student’s *t* test (for continuous variables) and the Fisher’s exact test or Chi-square test (for categorical variables). Post hoc power calculations were calculated using an online tool [15].

Univariate and multivariate logistic regression analyses were used to compare the cumulative primary endpoint between the groups. Covariates were selected if judged to be clinically meaningful. For the multivariate analysis, these were: age, disease duration, MGFA class, indication for CT scan, and concomitant acute disease. Subgroups of patients reaching the primary endpoint were compared using the Fisher’s exact test. Time to primary endpoint was compared using the log-rank test and Kaplan–Meier curves were calculated with censoring after 30 days. Binominal 95% confidence intervals were calculated using Clopper–Pearson intervals. A *P* value of ≤0.05 was considered statistically significant.

## Results

The baseline characteristics (Table 1) for most variables were well matched between patients with contrast-enhanced CT studies and unenhanced CT studies. The two groups differed, however, in the indication for the CT study and the body region scanned as well in the presence of an acute concomitant disease.

Scans of chest and abdomen were performed more often with contrast agents in comparison with scans of the head and other regions. Indications for the scans in the contrast-enhanced group were more often dyspnea and other, non-neurological, acute symptoms, and concomitant non-neurological acute diseases were accordingly more frequent in the contrast-enhanced group. The type of contrast agents could not be extracted retrospectively from the available data in 61.6% of patients, Jopamiro 300 was used in 24.7%, Jopamiro 370 in 2.7%, and Iomeron 400 in 11% of patients. The mean dose of administered ICA in the contrast-enhanced group was 101.7 mL (SD 22.4 mL) and 103.3 mL (SD 18.7 mL) in patients reaching the primary endpoint. The volume could not be extracted in one patient.

Nine patients (12.3%) in the contrast-enhanced and two patients (3.8%) in the unenhanced CT group reached the primary endpoint of worsening of myasthenic symptoms within 30 days of the scans (Table 2). We did not find a statistically significant difference for the chances of this event between the two study groups (Table 2), though it should be noted that the sample size only afforded us a power of 0.37 for the detection of an 8.5% difference between the groups at a significance level of 0.05.

**Table 2:** Results

	Contrast-enhanced CT scans (*N* = 73)	Unenhanced CT scans (*N* = 52)	*P* value**
Acute reaction	1 (1.4%)	NA	NA
Primary endpoint	9 (12.3%); 95% CI 5.8––22.1%*	2 (3.8%); 95% CI 0.5–13.2%*	Univariate analyses: *P* = 0.12 (OR 3.52, 95% CI 0.73–17.0)
Subtypes of endpoint			
Severe (death or myasthenic crisis)	6 (8.2%) (4 myasthenic crisis, 2 deaths)	0	0.04^‡^
≥1 increase in MGFA class but not myasthenic crisis or death)	3 (4.1%)	2 (3.8%)	1.00
Time to primary endpoint	11.1 days (SD 8.6)	13 days (SD 1.4)	0.10

Primary and secondary endpoints, as well as time to primary endpoint

*MGFA* Myasthenia Gravis Foundation of America, *OR* odds ratio, *CI* confidence interval, *SD* standard deviation

* Binominal 95% confidence intervals were calculated using Clopper–Pearson intervals

** *P* values were obtained with univariate or multivariate (adjusted for age, disease duration, MGFA class, indication, and concomitant acute disease) logistic regression analyses for the primary endpoint, with the Fisher exact test for subgroups comparison and the log-rank test for time to primary endpoint

^‡^Statistically significant

Multivariate analysis revealed significant effects of age and MGFA class for the occurrence of the primary endpoint. The administration of ICAs had no effect in this analysis (Table 2).

**Fig. 2:**
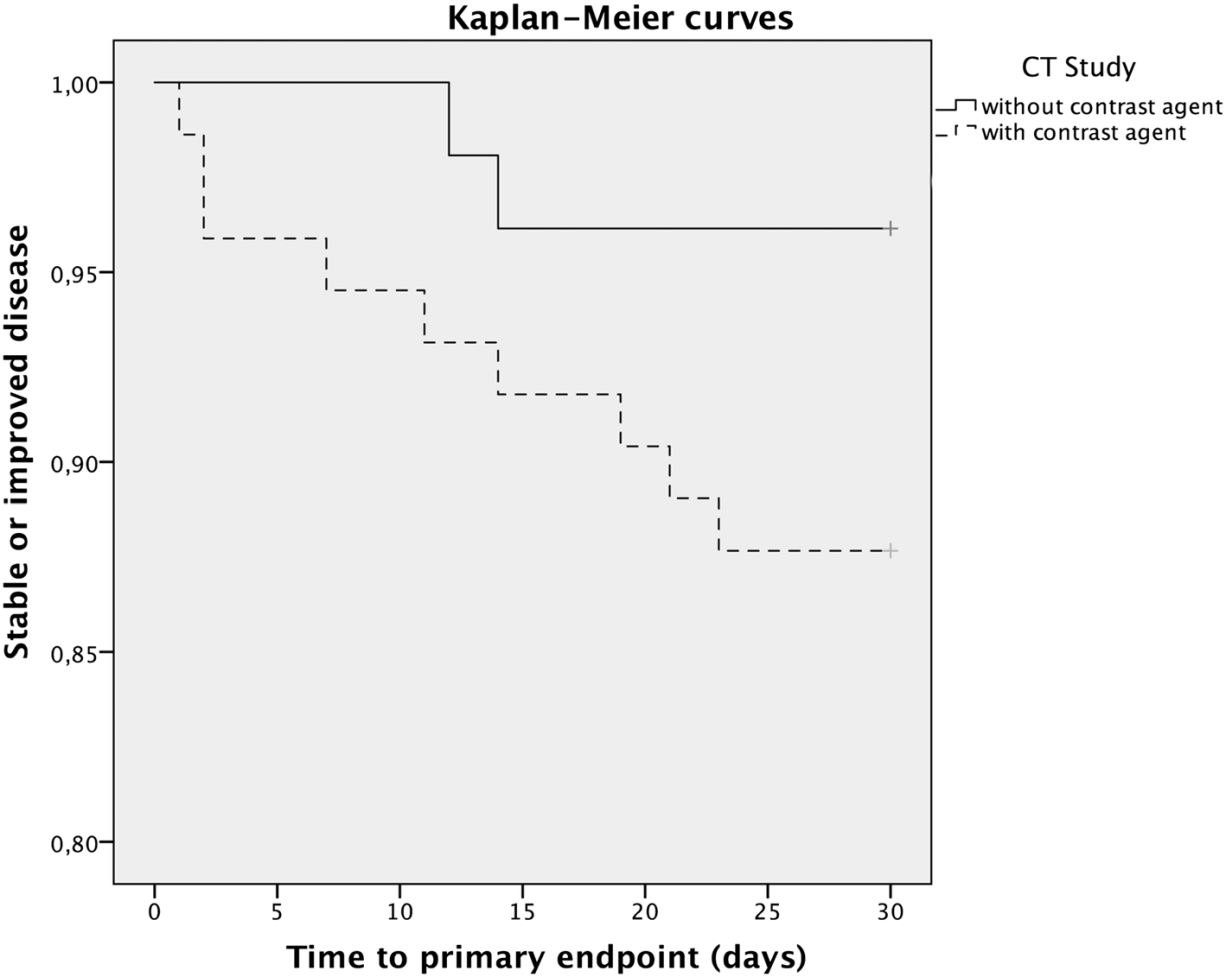
Kaplan–Meier curves for the primary endpoint of patients undergoing contrast-enhanced and unenhanced CT scans

The mean time to worsening within 30 days did not differ significantly between the two study groups and was 11.1 days for patients with contrast-enhanced CT studies and 13 days in the control group (Table 2; Fig. 2).

We detected only one patient who reached the secondary endpoint of an acute worsening of symptoms immediately after administration of a contrast agent documented in the radiological report. The event was described as a transient increase of preexisting dyspnea for which CT scanning was performed.

The subgroup analysis of patients having reached the primary endpoint was limited because of the small absolute numbers. Still, a comparison between the two groups regarding the proportion of patients experiencing a severe worsening (i.e., myasthenic crisis or death) reached nominal significance suggesting a greater risk for patients in the ICAs group. In fact, a severe exacerbation of symptoms was observed for six patients in the contrast-enhanced CT group but not at all in the control group (Table 2). Of these six patients, two died and four developed a myasthenic crisis.

### Case summaries of patients reaching the primary endpoint

In the control group, two patients showed clinical worsening 14 and 12 days after unenhanced CT of the chest was performed for thymus evaluation in new onset MG. Clinical deterioration was attributed to the natural course of the disease under insufficient therapy.

To differentiate between ICA being a possible cause of the clinical worsening or just an innocent, merely associated factor in a developing deterioration for other reasons, a detailed clinical analysis of the nine patients in the contrast-enhanced group was performed (Table 3). All nine patients had antibodies against acetylcholine receptor. None of these patients was the contrast agent considered, on clinical grounds, a likely cause of the deterioration:

**Table 3:** Detailed description of patients with exacerbation of myasthenic symptoms

	Age; sex	MGFA pre CT	MGFA after CT	Region; ICA dose	Indication	Time to endpoint	Clinically likely cause of endpoint
1	77; f	IIB	IVB	Chest; 90 ml	New onset MG; thymus evaluation	23 days	Insufficient therapy (only cholinesterase inhibitors)
2	68; m	IIIA	V	Chest; 90 ml	New onset MG; thymus evaluation	7 days	Nightly aspiration and respiratory worsening during plasma exchange the following day
3	73; m	IIIB	V	Chest and neck; 90 ml	New onset MG; dysphagia	11 days	Respiratory insufficiency due to insufficient therapy (only cholinesterase inhibitors)
4	69; f	IVB	V	Chest; 120 ml	New onset MG; dyspnea	1 day	1000 mg Prednisolone 2 days and Diazepam the day before CT scanning
5	80; f	IIIA	Death	Chest and abdomen; 140 ml	Weight loss, reduced general state of health	2 days	Previously unknown metastasized central lung carcinoma; death due to respiratory insufficiency
6	72; f	V	Death	Chest and abdomen; 90 ml	Dyspnea	14 days	Small cell lung cancer encompassing nearly the entire right lung; death after extubation because of respiratory insufficiency due to pulmonary edema
7	66; f	MM-3	IIIB	Head and chest: 100 ml	Staging, hepatic tumor	21 days	Azathioprine discontinued. Infection of unknown etiology with necessity of different antibiotic therapies (vancomycin, meropenem, piperacillin/tazobactam)
8	58; f	IIIA	IVB	Abdomen; 120 ml	Perforation of common bile duct after ERCP	2 days	Perforation of the common bile duct during ERCP with necessity of surgical treatment
9	60; f	IIIA	V	Chest; 90 ml	Suspected tumor in X-ray	10 days	Previously unknown mediastinal diffuse large B-cell lymphoma. Pneumonia and pleural effusions
10	64; m	IIA	IVA	Chest, unenhanced	New onset MG, thymus evaluation	14	Progressive muscular exhaustion within the natural course of disease
11	64; f	IIIB	IVB	Chest, unenhanced	New onset MG, thymus evaluation	12	Natural course of the disease under insufficient therapy (only cholinesterase inhibitors)

Characteristics of patients with delayed clinical worsening within 30 days after contrast-enhanced CT studies (number 1–9) and after unenhanced CT studies (number 10–11)

*MG* myasthenia gravis, *MGFA* Myasthenia Gravis Foundation of America, *ERCP* endoscopic retrograde cholangiopancreatography, *MM* minimal manifestation, *ICU* intensive care unit

*Patient 1* MG was diagnosed in this 77-year-old woman with initially mild weakness of oropharyngeal muscles. Chest CT was performed for evaluation of thymic pathology and revealed no pathological findings. Aggravation of symptoms occurred 23 days after CT scan, most likely reflecting the natural course of the disease with insufficient therapy (only cholinesterase inhibitors at the time). After repeated plasmaphereses (PLEX) treatment and initiation of prednisolone therapy, the patient subsequently improved.

*Patient 2* Chest CT was performed in this 68-year-old man with new onset MG for thymus evaluation and was without relevant pathological findings. The patient initially presented with progressive weakness of limb and ocular and bulbar muscles. Cholinesterase inhibitors and repeated PLEX were started, but myasthenic crisis still developed (7 days after the CT scan), presumably as part of the natural course of the disease compounded by aspiration pneumonia. After high prednisolone treatment and subsequent intravenous immunoglobulins and eventually thymectomy, the patient improved.

*Patient 3* MG was newly diagnosed in this 73-year-old man with initially moderate weakness of oropharyngeal muscles. CT of the neck and chest was performed because of dysphagia and for evaluation of thymic pathology (without relevant pathological findings). Myasthenic crisis occurred 11 days after CT scan, most likely reflecting the natural course of the disease with insufficient therapy (only cholinesterase inhibitors at the time). The patient improved subsequently after repeated PLEX was performed and therapy with prednisolone as well as azathioprine was started.

*Patient 4* Chest CT was performed because of progressive dyspnea in this 69-year-old woman who additionally suffered from severe oropharyngeal and to a lesser extent limb weakness. She had received high-dose prednisolone (1000 mg) 2 days and diazepam the day before because of suspected cervical spine pathology at an orthopedic ward. One day after CT scanning, the patient had to be intubated because of respiratory insufficiency. MG was subsequently diagnosed and the patients improved after adequate therapy (cholinesterase inhibitors and prednisolone).

*Patient 5* CT of the chest and abdomen was performed in an 80-year-old woman with a known MG (moderate limb weakness at the time of CT) because of weight loss and a reduced general state of health. CT showed a previously unknown central lung carcinoma (stage 4) with widespread metastasis. Because of the extensive disease, no escalation of therapy was performed and the patients died due to respiratory insufficiency caused by the tumor 2 days after CT scan.

*Patient 6 A* CT scan of the chest and abdomen was performed in this 72-year-old woman with a known MG after intubation had become necessary because of progressive dyspnea. The CT revealed a small cell lung cancer encompassing nearly the entire right lung. After extubation, the patient died 14 days after the CT scan because of respiratory insufficiency due to pulmonary edema.

*Patient 7* This 66-year-old woman underwent a CT study of the head and chest for staging of a hepatic tumor. At the time of the CT scan, the patient showed only minimal manifestation of myasthenic symptoms. 21 days after the CT scan, the patient developed moderate weakness of oropharyngeal and limb muscles presumably due to the discontinuation of azathioprine and a concomitant severe septic infection of unknown origin that required different antibiotic therapies (vancomycin, meropenem, and piperacillin/tazobactam). The patient improved after therapy with IVIG and prednisolone.

*Patient 8* CT scan of the abdomen was performed in this 58-year-old woman with a known MG because of a perforation of the common bile duct during an endoscopic retrograde cholangiopancreatography procedure. Surgical treatment was necessary and the patient exhibited a transient worsening of myasthenic symptoms with severe weakness of oropharyngeal muscles 2 days after the CT scan most likely as a consequence of the abdominal complication and/or surgical intervention.

*Patient 9* This 60-year-old woman with established MG underwent a CT scan of the chest because of a suspected tumor. CT showed a previously unknown mediastinal diffuse large B-cell lymphoma. The patient was intubated 10 days after the CT scan because of respiratory failure due to pneumonia and pleural effusions.

## Discussion

In this study, we retrospectively investigated the occurrence of adverse events after administration of modern, low-osmolality CT-contrast agents in patients with myasthenia gravis. We ascertained only a single patient (1.4%) with an acute, transient probably anaphylactic reaction (dyspnea) occurring immediately after application of the contrast agent. This rate is within the range of the previous studies in patients with or without myasthenia gravis [4, 6].

The other main finding of the study was that 9 of 73 patients (12.3%) experienced a delayed worsening of myasthenic symptoms, i.e., they reached the primary endpoint of progressing by at least one grade in the MGFA classification within 30 days. This worsening can be said to have occurred in a temporal association with the ICA administration with a median delay of 11 days. The rate was higher in comparison with the control group of patients receiving CT scans without ICAs (3.8%), but the difference did not reach statistical significance in a low powered comparison. In a subgroup analysis, six of these nine patients (8.2% of all patients) developed a severe deterioration, i.e., a myasthenic crisis or died in comparison with none in the control group.

Temporal association does not necessarily imply the presence of a causal relationship. The figure of patients reaching the formal endpoints could include those in whom the clinical worsening was induced by ICA administration and others who were set to deteriorate independently of the contrast agent. The comparison with the control group (patients receiving no ICA) is not very informative for this purpose because of the inherent underlying selection bias. Patients receiving a contrast-enhanced CT scan more often suffered from acute concomitant (not neuromuscular) diseases, which was the reason for the contrast-enhanced CT study in the first place. An analysis of the individual patient charts confirms the above point in that, by clinical judgment, the worsening could be attributed in all patients with more likelihood to other causes than to the administration of the contrast agent. These other causes included an insufficient MG-specific therapy in a newly developing myasthenia gravis, changes in the immunosuppressive therapy, or rapidly emerging systemic diseases such as cancer. In support of this view, a multivariate analysis only identified older age and a higher MGFA class at baseline as significant risk factors for clinical exacerbation after CT studies but not the administration of contrast agents.

Limitations of this study are the mentioned selection bias for the enhanced and unenhanced CT scans and the relatively low patient numbers. The retrospective nature of this investigation entails the possibility that some adverse events might have been missed in some patients as we had to rely on electronic medical records. To minimize this effect, we only included patients with a sufficient clinical information available.

Finally, the exact characteristics of the used contrast agents could not be extracted retrospectively from the available data in all patients; therefore, we could not compare the potential side effects of different ICAs with each other.

Our figures are comparable to the only other literature reference reporting delayed exacerbation of myasthenic symptoms after low-osmolality intravenous contrast agents. Somashekar et al. [3] observed MG-related symptom exacerbation in 12.5% of patients within 45 days, though the median delay was only 2.5 days, shorter than the 11 days in our study. The authors could not separate any direct effect of the contrast agent versus that mediated by any concurrent diseases on the worsening of myasthenia gravis, a causal relationship between ICA and MG worsening remained, therefore, undetermined.

Summarizing our data and interpreting those of other authors, we conclude that an acute, non-MG-related adverse reaction is a rare event with a risk comparable to other patients. A delayed worsening of myasthenia gravis-related symptoms might occur in approximately 12% of patients after ICA administration. In most cases, this delayed reaction seems to be a purely temporal rather than a causative association. However, given the inevitable uncertainty regarding this analysis, a causative relationship cannot be excluded in all cases, a view which was only recently exemplified by the case report of a patient developing a myasthenic crisis hours after injection of a low-osmolality ICA [16].

Balancing risks and benefits, we argue that with justified indications for contrast-enhanced CT scans, ICAs should not be generally withheld from patients with myasthenia gravis. Further prospective studies are clearly necessary to evaluate the true risk of ICAs in patients with myasthenia gravis.

## Abbreviations

CI: Confidence interval
CT: Computed tomography
ICA: Iodinated contrast agent
MG: Myasthenia gravis
MGFA: Myasthenia Gravis Foundation of America
MuSK: Muscle-specific tyrosine kinase
OR: Odds ratio
SD: Standard deviation
